# Contextual factors associated with depression among Northern and Indigenous adolescents in the Northwest Territories, Canada

**DOI:** 10.1017/gmh.2021.21

**Published:** 2021-06-24

**Authors:** Carmen H. Logie, Candice L. Lys, Nina Sokolovic, Kayley Inuksuk Mackay, Holly Donkers, Amanda Kanbari, Sherri Pooyak, Charlotte Loppie

**Affiliations:** 1 Factor-Inwentash Faculty of Social Work, University of Toronto, Toronto, Canada; 2 Women's College Research Institute, Women's College Hospital, Toronto, Canada; 3 United Nations University Institute of Water, Environment & Health, United Nations University, Hamilton, Canada; 4 Fostering Open eXpression among Youth (FOXY), Yellowknife, Northwest Territories, Canada; 5 Ontario Institute for Studies in Education, University of Toronto, Toronto, Canada; 6 Canadian Aboriginal AIDS Network (CAAN), Vancouver, Canada; 7 School of Public Health and Social Policy, University of Victoria, Victoria, Canada

**Keywords:** Depression, adolescents, Arctic, food insecurity, dating violence, sexual orientation, gender, Canada

## Abstract

**Background:**

Persons in Arctic regions disproportionately experience depression. Knowledge gaps remain regarding factors associated with depression among adolescents in the Northwest Territories (NWT), Canada, where child and adolescent mental health hospitalizations are nearly 2.5 times the national rate. This study assesses correlates of depression among adolescents in the NWT.

**Methods:**

We conducted a cross-sectional survey with adolescents aged 13–18 in 17 NWT communities. We assessed associations between socio-demographic characteristics, dating violence, food insecurity and depression, measured with the 9-item Patient Health Questionnaire. We conducted ordered logistic regressions to assess associations with no, mild, or moderate/severe depression scores.

**Results:**

Participants (*n* = 399; mean age: 14.3, s.d.: 1.3) were mostly Indigenous (79%) and 45% reported food insecurity. Nearly half (47%) reported minimal/no depression symptoms, 25% mild symptoms and 28% moderate/severe symptoms. In multivariate analyses, participants who were cisgender women compared to other genders, sexually diverse *v*. heterosexual, and food insecure had double the odds of more severe depression symptoms. Among those dating, dating violence was associated with double the odds of moderate/severe depression symptoms.

**Conclusions:**

Findings support tailored interventions to address material (food insecurity), relational (dating violence) and symbolic (gender and sexual orientation norms) contextual factors associated with depression among adolescents in the NWT.

## Background

Advancing mental health among adolescents in Arctic regions is a global priority (Collins *et al*., 2017; Trout and Wexler, 2020). Arctic populations are disproportionately impacted by mental health challenges compared with non-Arctic counterparts (Lehti et al., 2009; Collins *et al*., 2017; Trout and Wexler, 2020). There are longstanding challenges realizing optimal social determinants of health in Arctic regions, particularly housing, food security and healthcare access (Collins *et al*., 2017). Social determinants of health may play a particularly important role in shaping psychosocial stressors in the Arctic. For instance, crowded housing conditions among Inuit in Greenland were associated with poorer mental health, and this was exacerbated among women (Riva *et al*., 2014). Among Inuit youth in Nunavik, Canada, lower income and challenges finding animals to hunt were associated with poorer mental wellness, whereas cultural pride and positive social interaction were protective factors linked with improved mental wellbeing (Gray *et al*., 2016). Understanding factors associated with depression among Arctic youth is especially relevant during the COVID-19 pandemic, which may exacerbate pre-existing mental health disparities (Polsky and Gilmour, 2020; The Arctic Council, 2020).

Among adolescents, depression is one of the most common mental health challenges and a suicide risk factor, with onset usually in mid-to-late adolescence (Petito *et al*., 2020). It is critical to halt psychological distress and promote wellbeing among adolescents. Chronic psychological stress among adolescents has harmful impacts on neurobiological systems that are connected with emotional and behavioral regulation across the lifecourse (De Bellis, 2001). Without treatment, depression can harm educational and social outcomes, and may contribute to other health concerns such as smoking and substance use (Petito *et al*., 2020). Depression usually impacts youth during mid-to-late adolescence, hence identifying illness and early intervention can reduce the likelihood that adolescents will develop severe depression or other psychosocial challenges (Petito *et al*., 2020). There are concerns that depression may be increasing among adolescents over time in North America, and in turn there may be an elevated number of young people with untreated depression (Mojtabai *et al*., 2016; Lu, 2019).

In systematic reviews, food insecurity is associated with an increased risk of depression (Pourmotabbed *et al*., 2020), as is intimate partner violence (Beydoun *et al*., 2012; Devries *et al*., 2013), both disproportionately affecting persons in the Northwest Territories (NWT), Canada (Moffitt and Fikowski, 2017; Tarasuk and Mitchell, 2020). Food insecurity refers to a lack of access to sufficient quantity and quality of food (Pryor *et al*., 2016). Rates of food insecurity in Canada are higher among Indigenous people than non-Indigenous counterparts and higher in Northern Canada than the country as a whole (Subnath, 2017). Approximately one in six people in the NWT and Yukon experience food insecurity (Tarasuk *et al*., 2019). A study in Nunavik reported that severe household food insecurity in adolescence was associated with the concurrent symptoms of depression and withdrawn attitude, and adolescents who experienced persistent household food insecurity during childhood and adolescence reported increased depression and anxiety (Bradette-Laplante *et al*., 2020). A national population-based study with the Canadian Community Health Survey found that severe food insecurity was consistently associated with increased mortality from suicide (Men *et al*., 2020). Between 2017 and 2018, rates of household food insecurity were 15.9% in the NWT, 1.8-fold higher than that of the national average of 8.8% (Statistics Canada, 2020). Interpersonal violence among women in the NWT is 10-fold the national rate at 18.9 admissions per 1000, with rates of 15.6/1000 for youth aged 15–24 (Government of Northwest Territories, 2019).

Depression is particularly important to understand among Northern and Indigenous youth in the NWT, which has a population of approximately 44 469 persons; the proportion of youth under 15 years in the NWT is 22%, *v.* the national average of 16% (Government of Northwest Territories, 2016a). The NWT encompasses the second largest proportion of Indigenous residents in Canada, comprising 50% of the population and including Dene (33%), Inuit (11%) and Métis (6%) communities (Government of Northwest Territories, 2016a). Self-perceived mental health, rating one's mental health as good or excellent, was lower in the NWT (59%) than the national average (69%), and lower among Indigenous persons (48%) than non-Indigenous persons (72%) (Government of Northwest Territories, 2014). Mental health hospitalizations among children and youth in the NWT are almost 2.5-fold higher than the national rate (11.7 per 1000 population *v*. 4.7) and highest among those 15–17 years (23.3/1000 population) (Government of Northwest Territories, 2019).

Socio-historic and contextual determinants may explain some of these mental health trends. In particular, colonial violence practices such as state-sanctioned residential schools, forced assimilation and family separation produce intergenerational harms for Indigenous persons (Bjerregaard, 2001; Lehti *et al*., 2009), including changes in kinship structures and lasting effects of trauma and abuse (Chenhall and Senior, 2009; Kirmayer *et al*., 2011; Bellamy and Hardy, 2015). Many consider colonial practices ongoing in Canada, evidenced in the disproportionate involvement of Indigenous children in the child welfare system (Ma, 2020), racialized and sexualized violence underpinning the missing and murdered Indigenous women crisis (Razack, 2016), and overrepresentation of Indigenous people who are incarcerated (Chartrand, 2019).

Despite contextual challenges, there is individual and collective resilience among NWT communities and a focus on strengthening connection to culture, identities and land (Kirmayer *et al*., 2011; Government of Northwest Territories, 2016b; McCalman *et al*., 2020). Resilience is identified as a key domain of adolescent wellbeing, and includes the opportunity to build capacity and skills to manage adversity (Ross *et al*., 2020). Social ecological approaches to resilience view it as shaped in larger contexts (Ungar, 2011), and this lens has been applied to understand land, nature, community and culture as sources of resilience among Indigenous youth and youth in Northern regions (Allen *et al*., 2014; Gray *et al*., 2016; Hatala *et al*., 2020). Yet, there is a lack of research examining contextual factors associated with depression among adolescents in the NWT, which is required to inform tailored intervention strategies.

This analysis aims to address these knowledge gaps informed by a social contextual theoretical framework (Campbell and Cornish, 2012; Campbell *et al*., 2013; Gibbs *et al*., 2018). Rooted in Freire's focus on community engagement to understand the root causes of poor health and to transform power inequities (Freire, 1972), Campbell and Cornish (2012) conceptualized health enabling environments as ‘contexts which support and enable health-enhancing attitude and behaviour change’ (p. 848). There are multiple dimensions of health-enabling contexts, including: *symbolic* contexts that shape socio-cultural values and beliefs, including what groups are valued and respected, such as those reinscribed by gender norms; *material* contexts, referring to access to resources, such as food and financial security and *relational* contexts, which include social capital within communities, and social relationships between peers, families and others (Campbell and Cornish, 2012; Gibbs *et al*., 2018). The social and health inequities in the NWT discussed above regarding mental health, food insecurity and intimate partner violence, signal the utility of a social contextual approach in identifying how the wider social context shapes wellbeing among adolescents.

We examined *material contexts*, including access to resources, through assessing food insecurity and urban/rural location, as both food insecurity (Pourmotabbed *et al*., 2020) and remoteness (Mendez *et al*., 2013) have been associated with poorer mental health in prior research. We also examined *relational contexts*, referring to interpersonal interactions and social dynamics, by assessing dating violence, as this is a noted challenge among adult women in the NWT (Government of Northwest Territories, 2019) and is associated with poorer mental health among adolescents (Devries *et al*., 2013). To assess *symbolic contexts*, referring to social identities that are valued and devalued, we examined gender (Wiens *et al*., 2017) and sexual orientation (Russell and Fish, 2016) that are associated with increased likelihood of depression in varying contexts. Although these contextual factors have been linked with depression across diverse contexts and populations, there are knowledge gaps regarding how social contextual factors may be associated with depression in adolescents in the NWT, Canada. Such information can inform programs and policy. The study objectives were to assess prevalence and social contextual correlates of depression among adolescents 13–18 years old in the NWT, Canada.

## Methods

This study is part of a community-based research project with the Indigenous sexual health program Fostering Open eXpression among Youth (FOXY), developed for Northern and Indigenous girls and young women in the NWT, and Strengths, Masculinities and Sexual Health (SMASH), the counterpart developed for Northern and Indigenous boys and young men in the NWT. Aligned with community-based research principles (Israel *et al*., 2003; Flicker *et al*., 2015), the study was designed with Indigenous community partners in the NWT to address local youth needs and priorities, Indigenous community partners co-led the study and are included as co-authors, the community advisory board for FOXY and SMASH includes a majority of members who are Indigenous and from the NWT, Elders contributed to the study design, community collaborators contributed to the interpretation of findings and writing of this manuscript, and the community partner FOXY owns the data and leverages study findings to inform program development and acquire program funding. This was also a collaboration with the Canadian Aboriginal AIDS Network, a national network that leads a collective response for holistic wellness of Indigenous communities across Canada. Part of the ethics approval process with the Aurora Research Institute in the NWT involves gaining approval from all communities for this study, and schools were invited to participate in FOXY and SMASH and all schools that wished to participate were included.

### Sample

This community-based study included a non-random sample of secondary school students from 17 NWT communities [Aklavik (2020 population: *n* = 696), Whati (2020 population: *n* = 532), Fort McPherson (2020 population: *n* = 791), N'Dilo First Nations Community (2020 population: *n* = 200), Lutselk'e (2020 population: *n* = 330), Fort Liard (2020 population: *n* = 561), Fort Simpson (2020 population: *n* = 1258), Yellowknife (2020 population: *n* = 21372), Ulukhaktok (2020 population: *n* = 477), Fort Resolution (2020 population: *n* = 549), Behchoko (2020 population: *n* = 1983), Inuvik (2020 population: *n* = 3399), Tuktoyaktuk (2020 population: *n* = 989), Hay River (2020 population: *n* = 3793), K'atl'odeeche First Nation (2020 population: *n* = 325), Fort Smith (2020 population: *n* = 2586), Norman Wells (2020 population: *n* = 735)] in the 2018–2019 academic year. We included 399 persons in this study, representing 11.7% of the total youth in the NWT in this age group (GNWT, 2021). Out of 25 communities with junior and secondary schools in the NWT, 17 communities were purposively selected to participate. Each school selected classrooms between grades 7 and 12 to participate in a sexual health workshop, and students in these classrooms were invited to participate. To identify the schools, FOXY conducted school-based outreach, worked with FOXY and SMASH peer volunteers, and schools also learned about FOXY and SMASH through word-of-mouth. As an Indigenous community-based agency in the NWT, FOXY and SMASH have built long-lasting relationships with schools across the NWT. There are no pan-Indigenous approaches to knowledge paradigms and protocols (Loppie, 2007; Lavallée, 2009), but researchers may have different responsibilities when working with Indigenous communities (Datta, 2018), including facilitating research processes that are appropriate, caring, respectful and leave positive impacts on the community, hence following both cultural and research protocols. Relationality, nurturing relationships with community, is a key approach of FOXY's work, and this includes building lasting relationships with schools over time and providing schools the agency to select classrooms to participate in workshops. Decolonization of research centers Indigenous ways of being and knowing the world, and may differ from Western approaches to objectivity in research sampling approaches (Simpson, 2014; Datta, 2018).

Inclusion criteria were ages 13 and older, attending classes in grades 7–12, able to provide informed consent and agreement to attend a sexual health workshop. The informed consent process, detailed elsewhere (Lys *et al*., 2016), involved multiple steps: (1) FOXY made plans to conduct the workshop at each school, the school selected classrooms to participate and notified students there were voluntary workshops they could take part in; (2) the school sent parents/guardians a reverse consent form at least 1 week prior to the workshop, this approach assumes consent unless parents or guardians complete the form; (3) on the first day of the workshop, students were informed about the workshop and survey and invited to participate, if they were interested, they completed assent forms. Reverse consent processes were undertaken to increase access to sexual health information and healthy relationship information among adolescents who may not have engaged parents, and aligns with research on youth sexual agency and empowerment to make decisions regarding research participation (Flicker and Guta, 2008). Sexual health workshop content and evaluations of these workshops have been described elsewhere (Lys *et al*., 2016, 2018); briefly workshops aim to improve STI knowledge, empowerment and safer sex-self efficacy, and employ multi-media arts-based methods to explore sexual health, healthy relationships, safer sex negotiation, gender and healthy communication. The current analysis is based on baseline cross-sectional survey results completed prior to workshop participation. Surveys included sociodemographic characteristics and mental health questions. Participants were compensated with a $25 CAD valued gift (clothing item).

### Measures

We assessed depression using the 9-item Patient Health Questionnaire (PHQ-9; Cronbach's alpha = 0.88, range = 0–27) (Kroenke *et al*., 2001). We followed scoring guidelines to categorize depression outcomes into: minimal to no depression (scores <5), mild depression (scores 5–9) and moderate or severe depression (scores 10 and above). Although the PHQ-2 has been used with this population before in the NWT (Logie *et al*., 2018*a*, *b*), the PHQ-9 is recommended as a depression screener for adolescents as it may have higher validity than the PHQ-2 (Allgaier *et al*., 2012). We measured resilience using the Child and Youth Resilience Measure (Liebenberg *et al*., 2012), an 11-item scale to assess individual, relational, communal and cultural resources (Cronbach's alpha = 0.89, range = 11–55), used in prior research with this population (Logie *et al*., 2018*a*, *b*). We assessed dating violence using the Revised Conflict Tactics Scale (Straus *et al*., 1996), a 20-item scale addressing psychological aggression, physical assault and sexual coercion that has commonly been used in studies with adolescents (Exner-Cortens *et al*., 2016). Due to the low frequency of different types of violence exposure (1–15%; with the exception of using a harsh tone of voice, 35%), violence scores were dichotomized based on whether or not participants had experienced any type of violence in intimate relationships.

Participants also reported on their age, gender (cisgender women, cisgender men, transgender and non-binary), sexual identity [lesbian, gay, bisexual, queer, two-spirit or other sexually diverse (LGBQ2S) or heterosexual], Indigenous identity, living in an urban (Yellowknife) or rural (outside Yellowknife) location. We assessed food insecurity with a single item utilized in prior research with this population (Logie *et al*., 2019) (‘how often do you go to sleep hungry because you do not have enough food to eat’, dichotomized to ever *v*. never).

### Analyses

Descriptive statistics were calculated to explore sample characteristics and the distribution of outcome variables. To assess whether the prevalence of depression varied across sociodemographic characteristics, we used chi-squared (χ^2^) tests for independence with binary outcome indicators and one-way analyses of variance for continuous variables.

All variables that were significantly associated with depression outcomes in bivariate analyses (*p* < 0.05) were then included in ordered logistic regression models to assess factors associated with the outcome of no, mild or moderate/severe depression. Results are reported as odds ratios, with corresponding marginal probabilities. Marginal probabilities were calculated based on the entire sample for all variables except for dating violence. Probabilities were calculated for the average participant: a 14-year-old Indigenous, heterosexual, food-secure cisgender young woman living in a rural region. Since only two-thirds had any dating experience, models including dating violence were run separately. To better understand correlates of mild depression, we also conducted binary logistic regression models with mild *v*. no depression as the outcome. Due to low rates of missing data, all analyses were conducted using list-wise deletion in Stata version 15 (StataCorp., 2017).

### Ethics approval

All procedures were approved by the Research Ethics Board of the University of Toronto (31602) and Aurora Research Institute (16410).

## Results

### Demographics

This analysis included 397 participants ranging from 13 to 18 years old (*M*_age_ = 14.3, s.d. = 1.3). Approximately half (47%, *n* = 186) of the sample reported minimal to no depression symptoms, whereas 25% (*n* = 100) reported mild depression symptoms and 28% (*n* = 111) reported moderate or severe depression symptoms; there was no missing data for this outcome. Sample characteristics are provided in Table 1.

**Table 1:** Participant social and demographic characteristics

	*N* (%) or mean (s.d.)	Missing
Age	14.3 (1.3)	0
Gender		
Cisgender women	198 (50%)	0
Cisgender men	191 (48%)	
Transgender or other gender category	8 (2%)	
Ethnicity		
Indigenous	312 (79%)	1
Non-Indigenous	84 (21%)	
Urbanicity		
Urban (Yellowknife)	66 (17%)	4
Rural (Other place in NWT)	327 (83%)	
Sexuality		
Sexually diverse (LGBQ2S)	58 (15%)	1
Heterosexual	338 (85%)	
Food security		
Food insecure	179 (45%)	3
Food secure	215 (55%)	
Dating violence		
Experienced violence	125 (51%)	154
No violence	118 (49%)	
Resilience score	39.6 (9.5)	6

### Factors associated with depression

Descriptive analyses revealed that, with the exception of living in an urban or rural region and resilience, all factors tested were associated with depression. On average, participants who identified as cisgender women, Indigenous, LGBQ2S, food insecure, having experienced dating violence and older were over-represented in the higher severity depression categories (see Table 2). Each of these factors was independently associated with increased odds of moderate/severe depression, compared to mild, minimal or none (see Table 3).

**Table 2:** Analysis of social and demographic factors across participant depression score categories

	Depression category				
	Minimal/none	Mild	Moderate/severe	χ^2^/*F*	*p* value
Distribution of social and demographic indicators by depression category^a^					
Binary variables					
Cisgender women (*v*. cisgender men and other genders)	67 (36%)	52 (52%)	79 (71%)	34.60	<0.0001
Indigenous (*v*. non-Indigenous)	135 (73%)	77 (77%)	100 (90%)	12.42	0.002
Urban (*v*. non-urban)	35 (19%)	19 (19%)	12 (11%)	3.79	0.15
LGBQ2S (*v*. heterosexual)	21 (11%)	10 (10%)	27 (24%)	11.63	0.003
Food insecure (*v*. food secure)	56 (31%)	54 (54%)	69 (62%)	31.73	<0.0001
Dating violence (*v*. no violence)	47 (41%)	30 (52%)	48 (69%)	13.37	0.001
Continuous variables					
Age	14.24 (1.23)	13.99 (1.07)	14.62 (1.40)	7.00	0.001
Resilience	39.12 (10.97)	41.41 (7.59)	38.65 (8.14)	2.60	0.08
Marginal probabilities and 95% confidence interval by depression category					
Gender					
Women (cisgender)	0.45 (0.35–0.54)	0.29 (0.24–0.34)	0.27 (0.19–0.34)		
Men/transgender/other gender	0.65 (0.57–0.73)	0.21 (0.16–0.26)	0.14 (0.09–0.18)		
Age					
13-year old	0.46 (0.35–0.57)	0.28 (0.23–0.34)	0.25 (0.17–0.34)		
16-year old	0.41 (0.30–0.52)	0.29 (0.24–0.34)	0.29 (0.20–0.39)		
Ethnicity					
Indigenous	0.45 (0.35–0.54)	0.29 (0.24–0.34)	0.27 (0.19–0.34)		
Non-Indigenous	0.57 (0.43–0.70)	0.25 (0.18–0.32)	0.18 (0.10–0.27)		
Sexuality					
LGBQ2S	0.27 (0.15–0.40)	0.29 (0.23–0.35)	0.44 (0.28–0.59)		
Heterosexual	0.45 (0.35–0.54)	0.29 (0.24–0.34)	0.27 (0.19–0.34)		
Food Security					
Insecure	0.27 (0.20–0.34)	0.29 (0.24–0.34)	0.44 (0.36–0.53)		
Secure	0.45 (0.35–0.54)	0.29 (0.24–0.34)	0.27 (0.19–0.34)		
Dating Violence					
Yes	0.32 (0.19–0.45)	0.30 (0.23–0.37)	0.38 (0.24–0.52)		
No	0.50 (0.36–0.63)	0.28 (0.21–0.35)	0.23 (0.13–0.33)		

a Results in these columns are reported as *N* (%) for the number of individuals in that depression category who identify as women, Indigenous, etc. for binary variables or the mean (s.d.) of the outcome in each category for continuous variables.

**Table 3:** Ordered and binary logistic regression models assessing depression categories

			Entire sample		Dating sub-sample	
	Odds ratio	95% CI	Adjusted odds ratio	95% CI	Adjusted odds ratio	95% CI
Ordered logistic regression model assessing depression categories (none/minimal, mild, moderate/severe)						
Age	1.16*	1.00–1.35	1.07	0.91–1.25	1.11	0.91–1.35
Gender (woman)	3.09**	2.11–4.53	2.31**	1.55–3.45	2.86**	1.69–4.86
Indigenous	2.16**	1.35–3.45	1.62	0.98–2.68	1.96	0.94–4.08
LGBQ2S	2.13**	1.25–3.64	2.14**	1.21–3.78	3.25**	1.45–7.27
Food insecure	2.92**	2.00–4.28	2.19**	1.47–3.26	1.65	0.98–2.76
Dating violence	2.41**	1.49–3.91	NA	NA	2.11*	1.25–3.54
Binary logistic models assessing mild depression outcomes *v.* no or minimal depression						
Age	0.83	0.67–1.03	0.80	0.63–1.01	0.83	0.63–1.10
Gender (woman)	1.92*	1.17–3.15	1.71*	1.01–2.88	2.56**	1.29–5.08
Indigenous	1.24	0.70–2.19	1.07	0.58–1.97	1.25	0.53–2.97
LGBQ2S	0.88	0.40–1.96	0.76	0.33–1.79	0.71	0.20–2.51
Food insecure	2.66**	1.61–4.40	2.44**	1.45–4.12	1.58	0.81–3.10
Dating violence	1.55	0.82–2.93	NA	NA	1.71	0.87–3.36

*p < 0.05, **p < 0.01

In multivariate analyses, only some of these factors were significant. All else being equal, the odds of a more severe type of depression were approximately double for: cisgender young women compared to other genders (cisgender young men, transgender or non-binary youth); LGBQ2S compared to heterosexual participants; food insecure compared to food secure participants (Table 3). Among those in dating relationships, reporting dating violence was associated with more than double the odds of moderate/severe depression, rather than mild, minimal or none (Table 3).

Notably, only certain factors were associated with increased mild depression relative to minimal or no depression (Table 3). Holding all else equal, the likelihood of experiencing mild depression was higher for cisgender young women than cisgender young men, and more than double (2.44-fold higher) for food insecure individuals compared to food secure counterparts. Considering only participants with dating experience, being a cisgender young woman was the only factor associated with mild depression. That is, all else being equal cisgender young women had 2.5 greater odds of experiencing mild depression than cisgender young men.

## Discussion

Findings reveal moderate to severe depression symptoms among 28% of this sample of adolescents in 17 NWT communities. These findings signal that addressing depression among NWT adolescents is important to advance wellbeing, and that there are intervenable social contextual factors in the environment that may contribute to improved mental health (Erskine *et al*., 2015; Ross *et al*., 2020). We address knowledge gaps regarding multi-level factors that increase vulnerability to depression among Arctic adolescents, including food insecurity, teen dating violence and gender and sexual identity related stressors. Scant research has addressed lesbian, gay, bisexual and transgender (LGBT) health in the Arctic, thus this is a significant contribution (Logie and Lys, 2015; Logie *et al*., 2019). We also highlight heterogeneity in factors that contribute to moderate–severe depression compared with minor depression, which itself is a risk factor for major depression (Rowe and Rapaport, 2006). The eight countries with approximately 4 million residents in the Arctic region (Larsen and Fondahl, 2015) share histories of colonization of Indigenous peoples, an underrepresentation in research – particularly among adolescents, and insufficient healthcare and transportation infrastructure, rendering findings relevant to other Northern, remote and Indigenous communities in global health research (Allen *et al*., 2011, 2014; Trout and Wexler, 2020).

We found strong associations between food insecurity and depression, aligning with the evidence base that food insecurity not only has nutrition-related harms for youth but can also impact mental health (Jones, 2017). For instance, food insecurity produces stress responses due to the uncertainty of acquiring adequate food, feelings of shame and powerlessness, and awareness of socio-economic disparities (Jones, 2017). Food insecurity is a longstanding challenge in Arctic regions, including in Canada, that requires multi-level innovative strategies such as community-based initiatives like the Nunavut Food Security Coalition (Wakegijig *et al*., 2013), understanding how to increase access to country (traditional) food (Newell and Doubleday, 2020; Caughey *et al*., 2021), alongside policy change (Galloway, 2017).

Similar to prior research, we found higher depression prevalence among young women than young men (Wiens *et al*., 2017); this may be due to girls' increased exposure to violence, pressure to conform to gender norms that intensifies in adolescence and inhibits opportunities and choices, and gender differences in coping strategies and biologic stress responses (Nolen-Hoeksema and Girgus, 1994; Hyde and Mezulis, 2020). Our finding that adolescent violent victimization was associated with higher depression is consistent with prior research (Devries *et al*., 2013; Johnson *et al*., 2014) and signals the importance of understanding the root causes of this violence and preventing it to avoid poor social, educational and health outcomes (Miller *et al*., 2018). As mentioned earlier, the prevalence of interpersonal violence among women in the NWT is 10-fold than the national rate (Government of Northwest Territories, 2019). Teen dating violence prevention in the NWT can build partnerships with youth, Northern and Indigenous organizations, and academics and leverage cultural and community resources and strengths (Morris, 2016). Such programs can also address social contexts of isolation and poverty that exacerbate violence in the NWT (Faller *et al*., 2018), and apply a gender transformative approach to explore and shift gender and power imbalances among adolescents (Gibbs *et al*., 2015*a*; Kågesten and Chandra-Mouli, 2020).

Our finding that sexual minority youth have higher depression prevalence than heterosexual youth corroborates prior research on sexual stigma as a mental health stressor that is amplified by peer regulation of sexuality and gender norms in adolescence (Russell and Fish, 2016), and this social isolation and stigma may be exacerbated in small and rural contexts in the NWT (Logie *et al*., 2019). Conceptualizing sexual identity within *symbolic contexts* of stigma and discrimination that produce stress and subsequent health disparities aligns with the movement to de-pathologize LGBT people by identifying (and ultimately addressing) the root causes of social and health disparities in the social environment, such as in social ecological models (Baral *et al*., 2013), social determinants of health approaches (Logie, 2012), the minority stress model (Chakrapani *et al*., 2017) and structural approaches to stigma (Parker and Aggleton, 2003). Further research on global mental health can explore social contextual stressors and adaptive coping strategies among LGBT youth in rural, remote and Arctic regions.

Differences in depression between Indigenous and non-Indigenous participants became non-significant in multivariable analyses, pointing to the role of structural and social factors in shaping health. This finding could also be considered positive news in light of prior research that highlights the higher prevalence of severe mental health issues among Indigenous peoples in the circumpolar region (Fraser *et al*., 2015) – including the NWT (Government of Northwest Territories, 2014; Government of Northwest Territories, 2019) – compared with non-Indigenous counterparts. This may be due to cultural protective factors or systemic differences in the NWT. For instance, there has been a concerted effort to map mental health services in the NWT within a First Nations Mental Wellness Continuum that includes: *Culture as Foundation*, including land and culturally based programs; *Partners in Implementation*, engaging Indigenous communities to increase on the land programing and traditional healing programs and *Indigenous Social Determinants of Health*, which includes factors such as language, culture, land, employment and environmental stewardship (Elman *et al*., 2019). Arts and land-based programing for youth in the NWT may have positive mental health impacts (Ballantyne, 2014; Fanian *et al*., 2015; Lys, 2018). This aligns with the earlier discussion of Indigenous youth resilience nurtured by land, community, family and cultural connectedness (Allen *et al*., 2014; Gray *et al*., 2016; Hatala *et al*., 2020). Protective factors for depression among Indigenous adolescents in the Arctic are an area with limited empirical evidence that warrants further investigation.

Our study has limitations. The cross-sectional design and non-probability sampling reduce generalizability of findings to all youth in the NWT. Although this depression prevalence we noted in participants is higher than the national studies of the prevalence of major depressive episodes reported among 12–19 year-olds (pooled prevalence of 5.5%), including in the territories (5.7%) (Yukon, NWT and Nunavut) (Wiens *et al*., 2017), findings are not directly comparable as national estimates were drawn from the randomized Canadian Community Health Survey (CCHS). CCHS also did not differentiate between mild, moderate or severe depression, which makes the minor depression results difficult to compare. The high depression prevalence in this study may be related, in part, to the non-random sample and the possible biases associated with it. Our sample included 11.7% of the 13–18 year olds in the NWT (GNWT, 2021). Compared with the general population in the NWT, our sample: had a greater proportion of Indigenous participants (79% *v*. 51%); was more likely to live outside of Yellowknife (83% *v*. 49%) and had a similar gender breakdown (50% cisgender women, 48% cisgender men and 2% transgender persons *v*. 51% male and 49% female [we assessed gender and NWT statistics report sex]) (GNWT, 2021). Additionally we used a single-item measure of food insecurity which could be strengthened by more comprehensive measures (Coates *et al*., 2007). We did not assess peer support, which was linked with reduced depression in prior studies with adolescent girls in the NWT and could be a protective factor (Logie *et al*., 2018*a*, *b*). Future research can test other adolescent-specific violence measures (Exner-Cortens *et al*., 2016), including attitudes toward adolescent dating violence (Exner-Cortens *et al*., 2016). Exploring crowded housing in the NWT among adolescents and linkages to depression is another area for future research, as this was a significant risk factor in other Arctic regions (Riva *et al*., 2014).

Despite these limitations, our study can inform interventions for NWT adolescents that consider *material* (food insecurity), *relational* (dating violence) and *symbolic* (gender and sexual orientation norms) contexts (Campbell and Cornish, 2012). This contextual framing can include youth generated strategies to increase agency and reduce stress, in turn creating health-enabling environments. This information can contribute to the larger program of global mental health research in the Arctic (Trout and Wexler, 2020). For instance, our findings can inform innovative approaches to exploring Arctic youth wellbeing across contexts, such as the Circumpolar Indigenous Pathways to Adulthood (CIPA) study that explored life history narratives of youth from Northeastern Siberian Eveny, Northern Norwegian Sami, Northeastern Canada Inuit, Northwestern Alaska Inupiat and Southwestern Alaska Yup'ik (Allen *et al*., 2014). Allen *et al*. (2014) describe the salience of exploring youth resilience across circumpolar regions:

The narratives of youth coming of age in circumpolar communities hold potential to also tell a still-evolving story of cultural continuity wrested from disruptive colonial legacies. Youth experience can provide a lens through which to explore ways certain resilience processes might remain deeply patterned within traditional cultural practices, alongside with new emergent strategies representing innovations. (p. 602)

A syndemics approach could also be applied to explore synergies between social inequities (food insecurity, inequitable gender and sexual orientation norms) and health inequities (depression) (Singer *et al*., 2017). Such approaches hold the potential to produce multiple social and health benefits, for instance, increasing food security (Jones, 2017), transforming gender norms (Gibbs *et al*., 2015*b*) and reducing LGBTQ2S stigma (Logie *et al*., 2019) could improve life opportunities and equity for NWT adolescents while also reducing depression. Urgent attention is needed to address depression and its contextual correlates with NWT adolescents. It is imperative to understand and attend to the ongoing and long-term impacts of COVID-19 on Arctic youth mental health, particularly among youth with pre-existing mental health challenges that may be sustained or exacerbated by pandemic related anxiety, social isolation and school closures (Council, 2020; The Lancet Infectious Diseases, 2020; The Lancet Psychiatry, 2020; Gadermann *et al*., 2021).
